# Gene Expression Profiles in Paired Gingival Biopsies from Periodontitis-Affected and Healthy Tissues Revealed by Massively Parallel Sequencing

**DOI:** 10.1371/journal.pone.0046440

**Published:** 2012-09-28

**Authors:** Haleh Davanian, Henrik Stranneheim, Tove Båge, Maria Lagervall, Leif Jansson, Joakim Lundeberg, Tülay Yucel-Lindberg

**Affiliations:** 1 Division of Periodontology, Department of Dental Medicine, Karolinska Institutet, Huddinge, Sweden; 2 Science for Life Laboratory, Division of Gene Technology, School of Biotechnology, Royal Institute of Technology (KTH), Solna, Sweden; 3 Department of Periodontology at Skanstull, Stockholm County Council Sweden, Stockholm, Sweden; University of Toronto, Canada

## Abstract

Periodontitis is a chronic inflammatory disease affecting the soft tissue and bone that surrounds the teeth. Despite extensive research, distinctive genes responsible for the disease have not been identified. The objective of this study was to elucidate transcriptome changes in periodontitis, by investigating gene expression profiles in gingival tissue obtained from periodontitis-affected and healthy gingiva from the same patient, using RNA-sequencing. Gingival biopsies were obtained from a disease-affected and a healthy site from each of 10 individuals diagnosed with periodontitis. Enrichment analysis performed among uniquely expressed genes for the periodontitis-affected and healthy tissues revealed several regulated pathways indicative of inflammation for the periodontitis-affected condition. Hierarchical clustering of the sequenced biopsies demonstrated clustering according to the degree of inflammation, as observed histologically in the biopsies, rather than clustering at the individual level. Among the top 50 upregulated genes in periodontitis-affected tissues, we investigated two genes which have not previously been demonstrated to be involved in periodontitis. These included interferon regulatory factor 4 and chemokine (C-C motif) ligand 18, which were also expressed at the protein level in gingival biopsies from patients with periodontitis. In conclusion, this study provides a first step towards a quantitative comprehensive insight into the transcriptome changes in periodontitis. We demonstrate for the first time site-specific local variation in gene expression profiles of periodontitis-affected and healthy tissues obtained from patients with periodontitis, using RNA-seq. Further, we have identified novel genes expressed in periodontitis tissues, which may constitute potential therapeutic targets for future treatment strategies of periodontitis.

## Introduction

Periodontitis is a chronic inflammatory disease characterized by the destruction of periodontal tissue. This common disease, primarily initiated by periodontal pathogens, is an outcome of a complex interaction between periodontal microorganisms and the host inflammatory response [1]. The host response involves proinflammatory cytokines, chemokines, prostaglandins, Toll-like receptors and proteolytic enzymes, which have all been demonstrated to play an important role in the pathogenesis of periodontitis [2], [3].

Studies have been performed combining *in vivo* and *in vitro* approaches to identify genes responsible for periodontitis. To date, there are a few published microarray studies investigating the gene expression profile in periodontits. One microarray study reported no significant differences in gene expression at different pathological sites in patients with chronic and aggressive periodontitis [4], whereas Kim et al. [5] and Demmer et al. [6] showed a number of genes that were upregulated in periodontitis compared to healthy controls. In addition, Beikler et al. [7] demonstrated that in periodontitis sites, the expression of immune and inflammatory genes was down-regulated following non-surgical therapy. With regard to *in vitro* studies, gene expression profiling has been performed on gingival fibroblasts from inflamed and healthy gingival tissues, for a limited number of inflammatory markers, such as interleukin (IL)-1, IL-6, IL-8, tumor necrosis factor- α (TNF-α) and CD14 [8]. Furthermore, microarray analysis has also been performed on periodontal ligament cells and gingival keratinocytes [9], [10]. With regard to disease susceptibility at a genomic level, one genome-wide association study (GWAS) has been conducted in patients with aggressive periodontitis showing an association between aggressive periodontitis and intronic single nucleotide polymorphism rs1537415, which is located in the glycosyltransferase gene GLT6D1 [11].

Despite research investigating periodontitis gene expression profiles through microarray analysis, specific genes responsible for the disease have not yet been found. However, the recent development of massively parallel sequencing has provided a more comprehensive and accurate tool for gene expression analysis through sequenced based assays of transcriptomes, RNA-Sequencing (RNA-Seq). This method enables analysis of the complexity of whole eukaryotic transcriptomes [12] and studies comparing RNA-Seq and microarrays have shown that RNA-Seq has less bias, a greater dynamic range, a lower frequency of false positive signals and higher reproducibility [13], [14]. The aim of the present study was to investigate the general pattern of the gene expression profile in periodontitis using RNA-Seq. We also aimed to investigate the local variation in gene expression at site level, comparing periodontitis-affected and healthy gingival tissues obtained from the same patient.

## Materials and Methods

### Ethics Statement

The study was performed in accordance with the Declaration of Helsinki and the current legislation in Sweden and after approval from the Karolinska Institutet Ethical Research Board. The Regional Ethics Board in Stockholm approved the collection of the biopsies and informed consent was obtained from all patients.

### Collection of gingival tissue samples

A total of 10 nonsmoking individuals (20 biopsies), were included in the study. Four patients in the study group had other types of diseases: patient 2 was undergoing investigations for the disease sarcoidosis, patient 3 had diabetes type-2, patient 7 had a history of osteoarthritis and patient 10 was diagnosed with asthma. All participants were examined for periodontal disease and those with a tooth site demonstrating a probing depth ≥6 mm, clinical attachment level ≥5 mm and bleeding on probing were included in the periodontitis-affected group, according to the clinical parameters previously used as indicators of periodontitis [15], [16], [17]. During flap surgery, two adjacent gingival biopsies with identical clinical status were harvested from a periodontal pocket affected by periodontitis. The sizes of the specimens were approximately 2×2 mm, and included the connective tissue and the epithelium. In the same subjects, two adjacent gingival biopsies with identical clinical status and of about the same size were also obtained from a clinically healthy gingival pocket. Clinically healthy pockets were defined as sites with no gingival/periodontal inflammation, no bleeding on probing, a probing depth ≤3.5 mm and a clinical attachment level ≤3.5 mm. One of the biopsies from each site was stored in RNA Later (Applied Biosystems, USA) overnight at 4°C and thereafter stored at −80°C for subsequent RNA isolation. The second biopsy from each site was used for histological and immunohistochemical analysis.

### Hematoxylin-Eosin staining

Deparaffinized serial sections of gingival tissues were formalin fixed (4% neutral buffered formalin) and paraffin embedded. For assessment of orientation of the epithelium and connective tissue as well as the degree of inflammation, deparaffinized serial sections (4 µm) were prepared and sections of each biopsy were stained with Hematoxylin-Eosin (H&E). The degree of inflammatory cell infiltration was evaluated by three blinded observers, using a relative scale from 0 to 3, and statistical differences between periodontitis-affected and healthy sites were tested using the Wilcoxon signed-rank test.

### Immunohistochemical stainings in gingival tissue

For staining of the T cell marker CD3, interferon regulatory factor 4 (IRF4) and chemokine (C-C motif) ligand 18 (CCL18), gingival tissues were rinsed in phosphate buffered saline (PBS) with 0.1% Saponin (PBS-Saponin buffer) for 10 min. After an antigen retrieval procedure, 10 mM Tris, 1 mM EDTA (pH 9.0) for CD3 and 0.01 M Citrate acid (pH 6.0) for IRF4 and CCL18, sections were blocked in 1% H_2_O_2_ in PBS-Saponin for 60 min at room temperature (RT) for CD3 and for 45 min at RT for IRF4 and CCL18. Subsequently, tissues were rinsed in PBS-Saponin for 10 min and further treated with 3% bovine serum albumin (BSA) diluted in PBS-Saponin for 30 min at RT. The expression of CD3, IRF4 and CCL18 was investigated using CD3 polyclonal rabbit antibody (1 µg/ml, PBS-Saponin) from Dako Sweden AB (Stockholm, Sweden), IRF4 polyclonal rabbit antibody (0.5 µg/ml, PBS-Saponin) from Atlas antibodies (Stockholm, Sweden) and CCL18 polyclonal rabbit anti-human antibody (0.5 µg/ml, PBS-Saponin) from Sigma-Aldrich (St. Louis, MO, USA). Normal rabbit IgG from R&D systems (MN, USA) was used as negative control. After incubation with primary antibody, sections were blocked with 1% normal goat serum in PBS for 15 min. Afterwards, sections were incubated with a biotinylated secondary antibody provided in the Vectastain ABC-Elite Complex Kit (Vector labs, Burlingame, CA, USA) followed by application of the Elite ABC solution for 40 min at RT in the dark. Thereafter, sections were washed with PBS and the peroxidase activity was visualized with 0.3% (v/v) in DAB buffer containing 0.1% (v/v) H_2_O_2_. Finally, the slides were washed with distilled water, dehydrated through an ethanol series (70%, 95%, 99.9%) into xylene, mounted, and photographed using a light microscope. For CD3 stainings, the amount of positive cells was evaluated by three blinded observers, using a relative scale from 0 to 3, and statistical differences between periodontitis-affected and healthy biopsies were tested using the Wilcoxon signed-rank test.

### RNA extraction

RNA was extracted from gingival biopsies using steel-bead matrix tubes and a tabletop Fast-Prep homogenizer by two sequential centrifugations for 20 s at speed 6.5 (Qbiogene, Irvie, CA, USA). The RNA was purified on RNeasy Spin Columns (Qiagen, Valencia, CA, USA), treated with DNAse H to ensure degradation of DNA, and thereafter eluated in RNase-free water. The average RNA yield was 15.6 µg. RNA quality was assessed using the RNA 6000 NanoLabChip Kit of the Bioanalyzer system from Agilent Technologies (Santa Clara, CA, USA).

### Transcriptome sample preparation for sequencing

A total amount of 2–3 µg per sample was used as input material for the RNA sample preparations. All samples had RIN values above 8. The samples were bar-coded and prepared according to the protocol (Cat# RS-930-1001) from the manufacturer (Illumina, San Diego, CA, USA), as previously described by Stranneheim et al. [18]. All sample preparation reagents were taken from the Illumina mRNA Sample Preparation Kit or ordered from vendors specified in the mRNA sample preparation protocol, except for automation specific reagents: carboxylic acid beads used for precipitation; the ethanol and tetraethylene glycol (EtOH/TEG) and the Polyethylene Glycol and sodium chloride (PEG/NaCl) precipitation buffers.

### Clustering and sequencing

The clustering of the bar-coded samples was performed on a cBot Cluster Generation System using an Illumina HiSeq Single Read Cluster Generation Kit according to the manufacturer's instructions. The library preparations were sequenced on an Illumina HiSeq 2000 as single-reads to 100 bp. Two sequencing runs were performed according to the manufacturer's instructions where two and three lanes were used in the first sequencing and second sequencing run, respectively (Table S1). The runs generated a total of 402 million reads with an average of 15 million reads per sample that passed the Illumina Chastity filter; these reads were included in the study.

### Sequence analysis

All sequences were aligned to the human genome reference hg19 with TopHat [19], [20] version 1.1.4 and Samtools [21] version 0.1.8 using TopHat standard parameters except for parameters –solexa1.3-quals -p 8 –GTF Homo_sapiens.GRCh37.59.gtf. Annotations from Ensembl and RefSeq, downloaded from University of California, Santa Cruz (UCSC) Genome Browser, were used to assign features to genomic positions. Sequences aligned to the human genome were assigned to features and counted using HTSeq version 0.4.6 with parameters -m intersection-strict -s no -t exon. The R/Bioconductor package DESeq [22] was used to call differential gene expression on read counts generated by HTSeq and to perform hierarchical clustering of samples. All biological replicates for healthy and periodontitis-affected had R^2^ (Spearman) correlation of gene expression (read counts) above 0.92.

### Functional analyses of gene lists using WebGestalt

Analyses of gene categories and pathways was performed using the WEB-based Gene Set Analysis Toolkit v2 (WebGestalt) [23] with parameters: Id Type: Ensembl_gene_stable_id, Ref Set: Entrez Gene, Significance Level: *p*<0.05, Statistics Test: Hypergeometric, MTC: BH, Minimum: 2. KEGG analysis was used for pathway enrichment analysis and the Gene ontology (GO) category Biological process was used for the functional annotation analysis.

## Results

### Patients and gingival tissues

**Figure 1:**
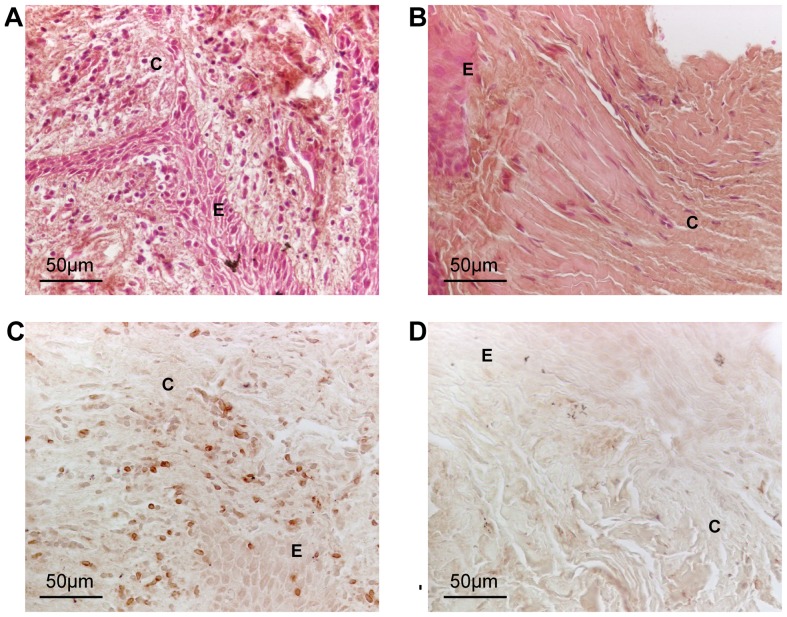
H&E and CD3-stained paraffin-embedded gingival biopsies obtained from one representative patient with periodontitis. A. H&E staining of inflammatory cells in periodontitis-affected sections. B. H&E staining of inflammatory cells in healthy gingival sections. C. Staining of the T-cell marker CD3 in periodontitis-affected sections. D. Staining of the T-cell marker CD3 in healthy sections. E, epithelium, C, connective tissue.

A total of 10 patients, six males and four females, with a mean age of 50±8, were included in the study. For each patient, a total of four gingival biopsies of about the same size were obtained from periodontitis-affected and healthy gingiva, with two biopsies from each site. Bleeding status, probing depth and degree of inflammation in the gingival tissues for each of the two gingival sites was recorded (Table 1). To assess the degree of gingival inflammation in the periodontitis-affected and healthy tissues, histological and immunohistochemistry staining was performed using H&E and anti-CD3 (Fig. 1). Scoring of the degree of inflammatory cell infiltration, assessed by H&E staining, and the amount of CD3 positive cells showed significantly higher inflammation in tissue from periodontitis-affected sites (*p<*0.01 for H&E and *p<*0.05 for CD3; Table 1).

**Table 1:** Patient characteristics and periodontal status.

Patient characteristics			Periodontitis-affected sites			Healthy sites		
Patient	Gender	Age	Probing depth (mm)	InflammationH&E (0–3)^a^^, ^ c	Inflammation CD3 (0–3)^b^^, ^ d	Probing depth (mm)	Inflammation H&E (0–3)^a^^, ^ c	Inflammation CD3 (0–3)^b^^, ^ d
1	M	52	7	3	2	3	2	0
2	M	45	8	1	1	3	1	1
3	M	52	7	3	3	3	1	1
4	F	47	7	3	3	3	1	2
5	F	37	7	2	2	3	1	1
6	M	59	7	2	3	3	2	1
7	F	66	7	2	-	3	0	-
8	M	48	6	2	2	3	0	0
9	M	42	8	3	1	2	1	1
10	F	54	6	2	1	3	1	1

a 0 = no evidence of inflammatory infiltration, 1 = slight inflammatory infiltration, 2 = moderate inflammatory infiltration and 3 = severe inflammatory infiltration.

b 0 = no CD3 positive cells, 1 = low amount of CD3 positive cells, 2 = moderate amount of CD3 positive cells, 3 = high amount of CD3 positive cells and – = not enough material to perform staining.

c Significant difference between periodontitis-affected and healthy sites (*p<0*.01).

d Significant difference between periodontitis-affected and healthy sites (*p<*0.05).

### RNA-Sequencing

**Figure 2:**
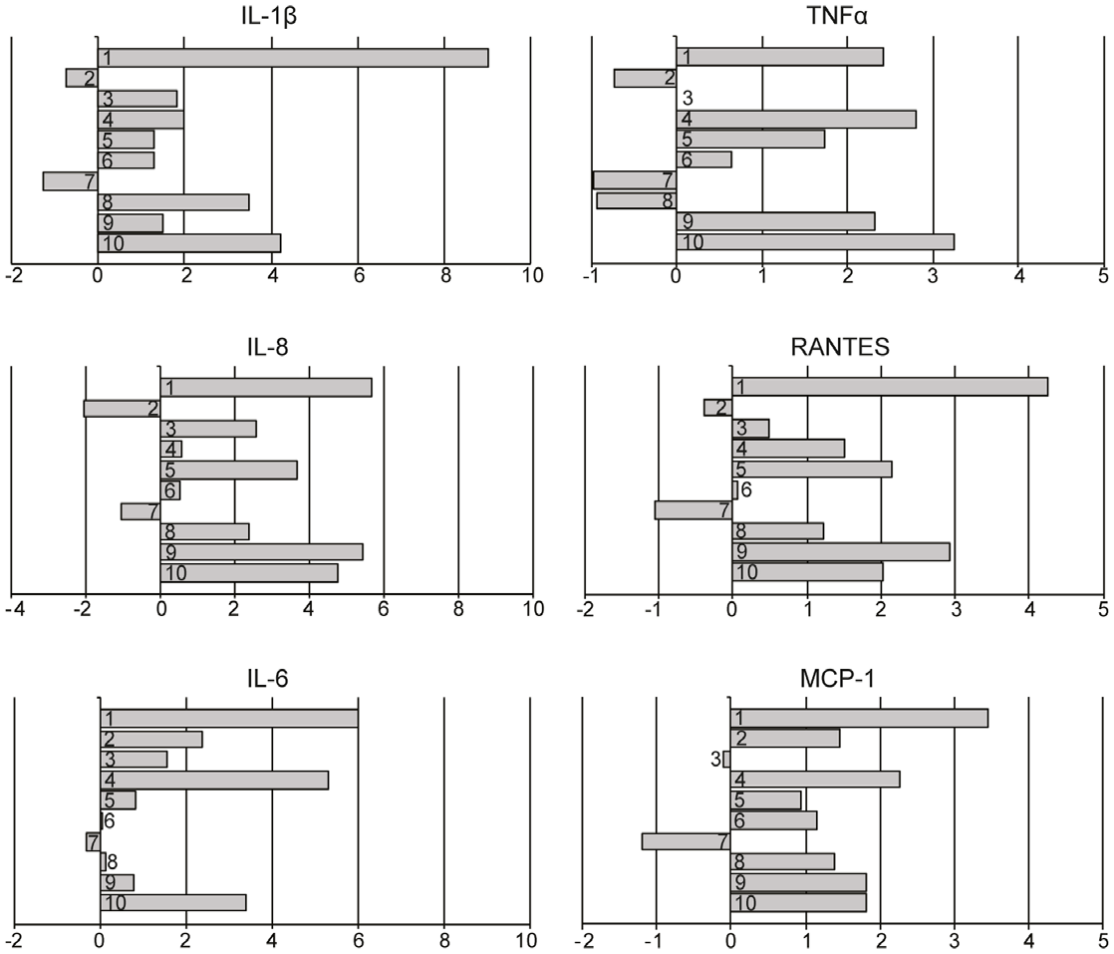
Expression of the inflammatory mediators in periodontitis-affected and healthy tissues obtained by RNA-seq. The bars show the expression (log_2_ fold change) pattern based on RNA-Seq reads of IL-1β, IL-6, IL-8, TNFα, RANTES and MCP-1.

We sequenced cDNA from 10 periodontitis-affected and 10 healthy gingival tissues, with an average of 15 million reads of 100 bp in length per sample. A pairwise approach, where each periodontitis-affected biopsy had a healthy counterpart from the same individual, was used to eliminate the background noise of individual-specific gene transcription, enabling acquisition of more relevant data from the cohort. Aligning the sequence reads against the human genome yielded a median of 68% of uniquely aligned reads across all samples. The expression pattern, based on RNA-Seq reads, of well-known inflammatory mediators IL-1β, IL-6, IL-8, TNFα, Regulated upon Activation, Normal T-cell Expressed, and Secreted (RANTES) and Monocyte Chemotactic Protein-1 (MCP-1) were analyzed in all the tissue samples. The expression (log_2_ fold change) of these mediators was shown to be higher in the majority of the periodontitis-affected gingival tissue compared to healthy gingival tissue from the same patient (Fig. 2).

### Distribution of gene transcripts between periodontitis-affected and healthy gingival tissues

**Figure 3:**
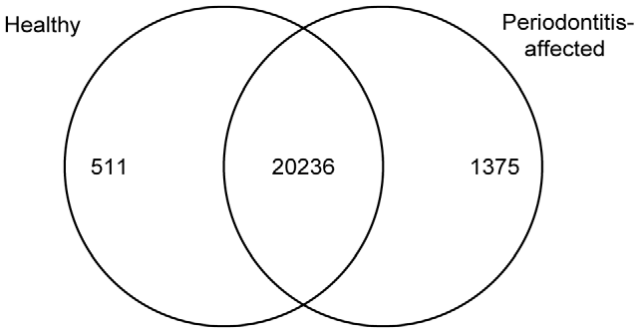
Venn diagram of mRNA transcripts. Venn diagram showing genes that were uniquely expressed in periodontitis-affected (1375) and healthy (511) gingival tissues. The intersection of the two circles refers to transcripts, which are expressed in both periodontitis-affected and healthy gingival tissues (20 236).

A total of 22 122 different mRNA transcripts were expressed in the periodontitis-affected and healthy gingival tissue samples. Among these transcripts, 1375 were unique to the periodontitis-affected tissue samples whereas 511 genes were uniquely transcribed in healthy gingival tissues (Fig. 3). KEGG enrichment analysis using WebGestalt [24] was performed among the unique genes for the periodontitis-affected and healthy tissues which revealed several regulated pathways indicative of inflammation for the periodontitis-affected condition (Table 2 and Table S1). In contrast, in the healthy gingival tissues, regulated pathways indicated a non-inflammatory profile among the unique genes, as demonstrated in Table 3 and Table S1.

**Table 2:** Enriched regulated (KEGG) biological pathways among unique genes in periodontitis-affected tissues.

Pathway	Total genes in pathway	Unique genes in pathway^a^	Adj *p* value^b^
Neuroactive ligand-receptor interaction	256	19	8.18e-10
Cytokine-cytokine receptor interaction	267	18	6.75e-09
Chemokine signaling pathway	190	10	0.0004
Intestinal immune network for IgA production	50	5	0.0014
Alanine, aspartate and glutamate metabolism	31	5	0.0022
Tyrosine metabolism	46	4	0.0103
Calcium signaling pathway	178	7	0.0160
Hedgehog signaling pathway	56	4	0.0161
Systemic lupus erythematosus	140	6	0.0168
Glycine, serine and threonine metabolism	31	3	0.0196
Jak-STAT signaling pathway	155	6	0.0229
Vascular smooth muscle contraction	115	5	0.0271
Arhythmogenic right ventricular cardiomyopathy (ARVC)	76	4	0.0293

a Lists of uniquely expressed genes within the enriched pathways can be found in Table S1.

b adj *p* value indicates the significance of the enrichment, (adj *p*<0.05).

**Table 3:** Enriched regulated (KEGG) biological pathways among unique genes in healthy tissues.

Pathway	Total genes in pathway	Unique genes in pathway^a^	Adj *p* value^b^
Neuroactive ligand-receptor interaction	256	11	8.18e-10
Glycolysis/Gluconeogenesis	62	3	6.75e-09
Calcium signaling pathway	178	4	0.0004
Gap junction	90	3	0.0014
Pyruvate metabolism	40	2	0.0022
Tryptophan metabolism	40	2	0.0103

a Lists of uniquely expressed genes within the enriched pathways can be found in Table S1.

b adj *p* value indicates the significance of the enrichment, (adj *p*<0.05).

### Clustering of biopsies

**Figure 4:**
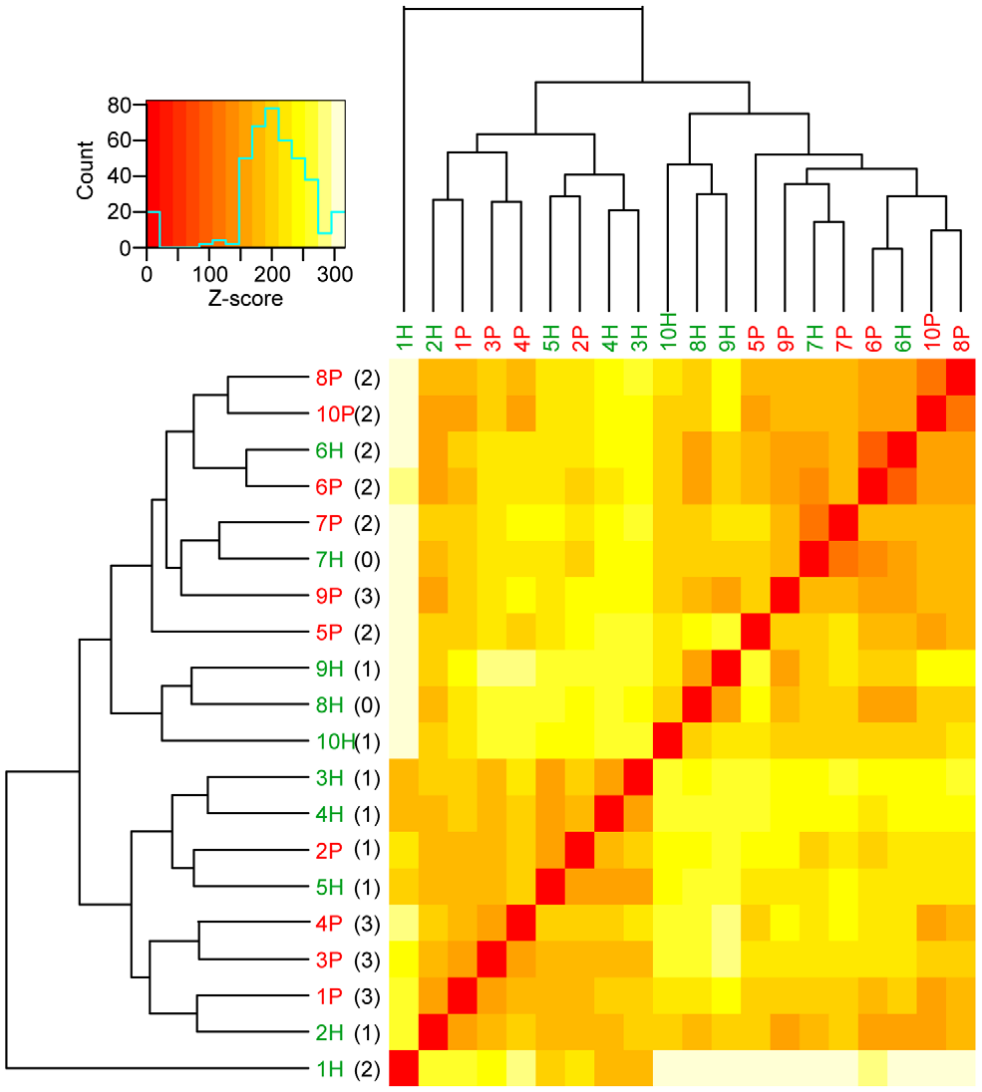
Clustering dendrogram and heatmap of periodontitis-affected and healthy biopsies. Clustering of all samples was based on gene transcripts with a median read above three times the background noise. The length of the branch between two biopsies and the colors of the heatmap correspond to degree of similarity between the gene expression profiles. Colors can be interpreted using the scale bar. Numbers in parentheses denote the inflammation scores of the biopsies after H&E histological evaluation.

Unsupervised hierarchical clustering was performed on all gene transcripts having a median read count above a cutoff level set to 0.3 read counts per feature, to exclude expression due to spurious transcription (Fig. 4). The gingival tissues from periodontitis-affected sites from different patients showed a more similar gene expression pattern than healthy gingival tissues from the same patient. Clustering according to individual, where the paired healthy and periodontitis-affected biopsies cluster together, was only observed for patient 6 and 7. However, the biopsies showed a general trend of clustering according to the degree of inflammation as assessed by H&E staining (Table 1), except for sample 7H, sample 2H and an outlier sample 1H, which clustered separately. There was also a trend of forming larger clusters depending on sequence run, but paired biopsies (periodontits-affected and healthy) from each patient were always analyzed in the same sequence run.

### Differential gene expression between periodontitis-affected and healthy gingival tissues

**Figure 5:**
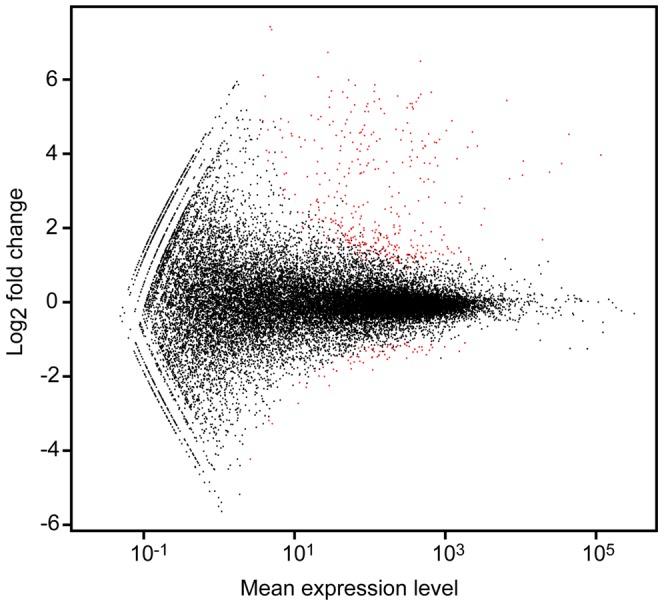
Volcano plot displaying differential expression. Differential gene expression (adj *p*<0.01) between periodontitis-affected and healthy gingival tissues. The *y* axis corresponds to the log_2_ fold change value (*M* value), and the *x* axis displays the mean expression value.

Differential gene expression between periodontitis-affected and healthy gingival tissues was analyzed using read counts for each gene with the DeSeq package [22]. The analysis revealed a total of 453 significantly (adj *p*<0.01) differentially expressed genes. Additional analyses of genes expressed in periodontitis-affected gingiva, showed that 381 genes were upregulated, whereas 72 genes were shown to be down-regulated (Fig. 5, Table S2).

### Gene Ontology enrichment analysis of differentially expressed genes

**Figure 6:**
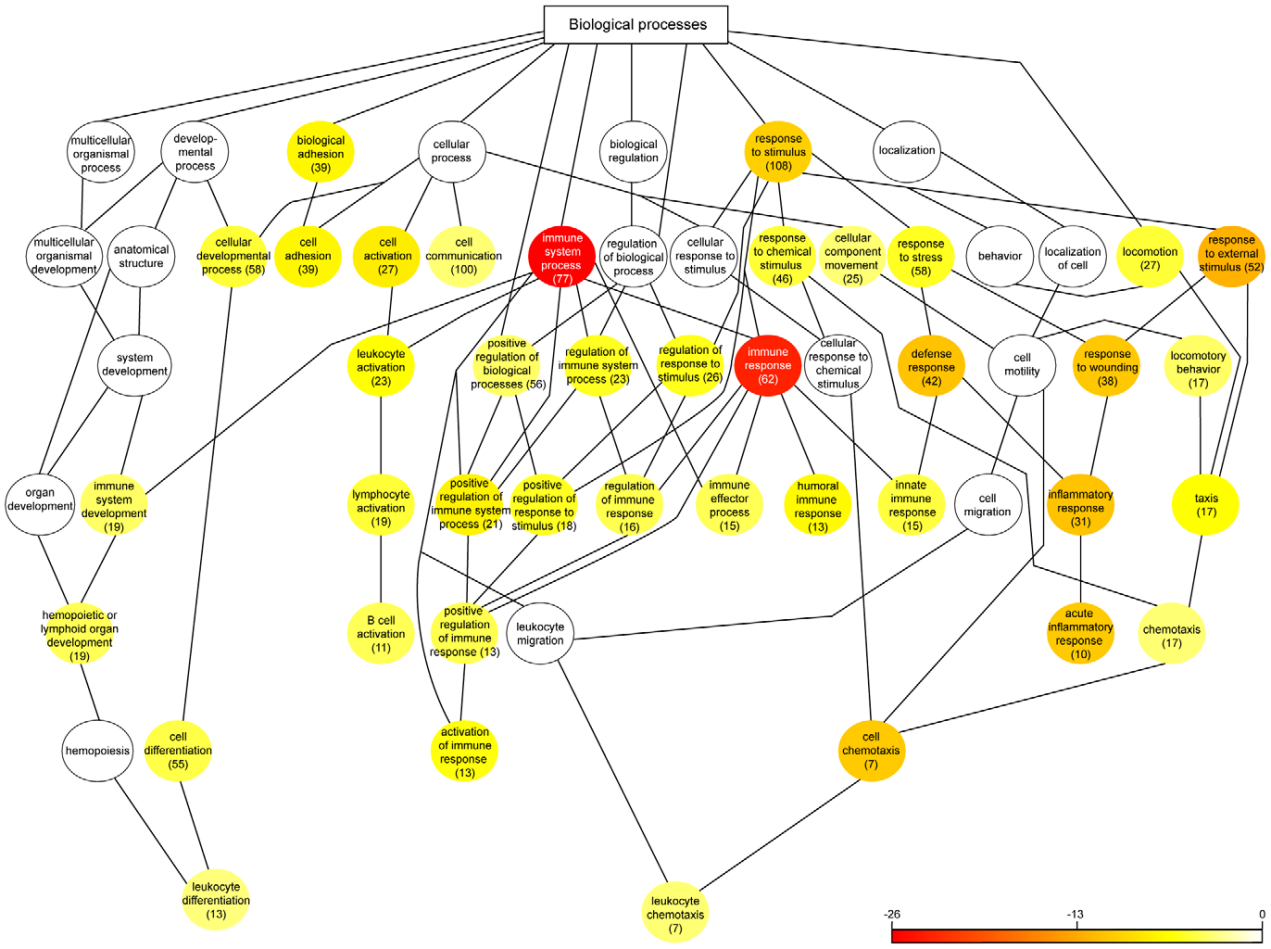
Gene ontology (GO) analysis of differentially expressed genes. All significant (*p*<0.05) Biological processes (GO categories) and their parent terms are shown. The color of each node illustrates the significance and can be interpreted using the scale bar, which displays the *p* value. Each node is also marked with the number of significantly regulated genes mapped to the GO category.

Investigation of functional associations of gene expression changes in the tissue samples was performed using WebGestalt. Gene ontology (GO) Biological process was used for enrichment analysis. Significant gene enrichments (*p*<0.05) as well as their parent terms are demonstrated in Fig. 6. Several GO categories were over-represented among genes differentially expressed in periodontitis-affected versus healthy gingival tissues. The categories were mainly indicative of immune and inflammatory responses. Further enrichment analysis regarding Molecular function and Cellular components are provided in the supplementary data (Table S3).

### Top 50 upregulated genes in periodontitis-affected gingival tissue

The top 50 significantly upregulated genes in periodontitis-affected gingival tissue with Unigene entry are displayed in Table 4 together with Ensemble ID, gene symbol, fold change, log_2_ fold change and *p* value. We investigated whether there were any available reports on the involvement of these genes in periodontitis or other chronic inflammatory conditions. Among the top 50 upregulated genes, we identified a number of candidate genes, which were not previously demonstrated to be involved in periodontitis but have been shown to be associated with other chronic conditions such as rheumatoid arthritis (RA). These candidate genes included FCRL5, adenosine monophosphate deaminase 1 (AMPD1), CCL18, tumor-necrosis factor receptor superfamily 17 (TNFRSF17) and leukocyte immunoglobin-like receptor, subfamily A (without TM domain) member 3 (LILRA3), and IRF4 which has shown to be involved in chronic inflammatory diseases such as RA and inflammatory bowel disease (IBD), (Table 5).

**Table 4:** Top 50 upregulated genes in periodontitis-affected tissue with Unigene entry.

Ensemble ID	Gene symbol	Description	Fold change	Log_2_ fold change	*p* value
ENSG00000188596	C12orf63	chromosome 12 open reading frame 63	69,15	6,11	9,54e-06
ENSG00000132704	FCRL2	Fc receptor-like 2	30,36	4,92	1,39e-10
ENSG00000143297	FCRL5	Fc receptor-like 5	25,24	4,66	5,24e-30
ENSG00000116748	AMPD1	adenosine monophosphate deaminase 1 (isoform M)	24,97	4,64	5,58e-05
ENSG00000187323	DCC	deleted in colorectal carcinoma	20,69	4,37	2,37e-09
ENSG00000137265	IRF4	interferon regulatory factor 4	20,10	4,33	1,50e-32
ENSG00000167077	MEI1	meiosis inhibitor 1	16,77	4,07	3,24e-16
ENSG00000101194	SLC17A9	solute carrier family 17, member 9	14,40	3,85	2,04e-14
ENSG00000122188	LAX1	lymphocyte transmembrane adaptor 1	14,28	3,84	3,83e-20
ENSG00000110777	POU2AF1	POU class 2 associating factor 1	14,12	3,82	9,81e-26
ENSG00000124256	ZBP1	Z-DNA binding protein 1	13,76	3,78	1,60e-14
ENSG00000170476	MGC29506	hypothetical protein MGC29506	13,33	3,74	1,20e-21
ENSG00000132185	FCRLA	Fc receptor-like A	12,18	3,61	2,47e-11
ENSG00000012223	LTF	lactotransferrin	12,09	3,61	8,54e-22
ENSG00000137673	MMP7	matrix metallopeptidase 7 (matrilysin, uterine)	11,37	3,51	8,33e-18
ENSG00000163534	FCRL1	Fc receptor-like 1	11,14	3,48	1,54e-05
ENSG00000177455	CD19	CD19 molecule	11,12	3,48	2,13e-08
ENSG00000061656	SPAG4	sperm associated antigen 4	11,09	3,47	1,62e-10
ENSG00000121895	TMEM156	transmembrane protein 156	11,00	3,46	2,35e-08
ENSG00000015413	DPEP1	dipeptidase 1 (renal)	10,93	3,45	8,81e-06
ENSG00000048462	TNFRSF17	tumor necrosis factor receptor superfamily, member 17	10,43	3,38	2,71e-08
ENSG00000169962	TAS1R3	taste receptor, type 1, member 3	10,42	3,38	2,28e-06
ENSG00000102096	PIM2	pim-2 oncogene	10,09	3,34	2,43e-23
ENSG00000183508	FAM46C	family with sequence similarity 46, member C	9,94	3,31	2,19e-24
ENSG00000168081	PNOC	prepronociceptin	9,75	3,29	1,83e-07
ENSG00000099958	DERL3	Der1-like domain family, member 3	9,45	3,24	2,26e-16
ENSG00000105369	CD79A	CD79a molecule, immunoglobulin-associated alpha	9,43	3,24	1,36e-18
ENSG00000189233	C8orf80	chromosome 8 open reading frame 80	9,03	3,17	2,42e-07
ENSG00000004468	CD38	CD38 molecule	8,75	3,13	7,57e-10
ENSG00000153789	FAM92B	family with sequence similarity 92, member B	8,21	3,04	2,95e-05
ENSG00000143603	KCNN3	potassium intermediate/small conductance calcium-activated channel, subfamily N, member 3	7,86	2,98	3,81e-07
ENSG00000007129	CEACAM21	carcinoembryonic antigen-related cell adhesion molecule 21	7,48	2,90	1,94e-05
ENSG00000170866	LILRA3	leukocyte immunoglobulin-like receptor, subfamily A (without TM domain), member 3	7,45	2,90	0,000111577
ENSG00000129988	LBP	lipopolysaccharide binding protein	7,33	2,87	7,78e-08
ENSG00000118308	LRMP	lymphoid-restricted membrane protein	7,24	2,86	7,08e-09
ENSG00000139193	CD27	CD27 molecule	7,21	2,85	4,63e-13
ENSG00000073849	ST6GAL1	ST6 beta-galactosamide alpha-2,6-sialyltranferase 1	7,11	2,83	4,37e-20
ENSG00000177272	KCNA3	potassium voltage-gated channel, shaker-related subfamily, member 3	7,07	2,82	4,64e-08
ENSG00000108405	P2RX1	purinergic receptor P2X, ligand-gated ion channel, 1	6,81	2,77	3,18e-05
ENSG00000026751	SLAMF7	SLAM family member 7	6,64	2,73	2,05e-16
ENSG00000124772	CPNE5	copine V	6,47	2,69	5,14e-10
ENSG00000132465	IGJ	immunoglobulin J polypeptide, linker protein for immunoglobulin alpha and mu polypeptides	6,41	2,68	6,97e-21
ENSG00000122224	LY9	lymphocyte antigen 9	6,39	2,68	1,71e-06
ENSG00000007312	CD79B	CD79b molecule, immunoglobulin-associated beta	6,28	2,65	1,89e-07
ENSG00000134873	CLDN10	claudin 10	6,17	2,63	2,82e-06
ENSG00000172578	KLHL6	kelch-like 6 (Drosophila)	6,16	2,62	1,98e-11
ENSG00000196549	MME	membrane metallo-endopeptidase	6,01	2,59	2,29e-16
ENSG00000006074	CCL18	chemokine (C-C motif) ligand 18 (pulmonary and activation-regulated)	6,00	2,59	5,68e-10
ENSG00000173432	SAA1	serum amyloid A1	5,91	2,56	7,97e-10
ENSG00000159618	GPR114	G protein-coupled receptor 114	5,89	2,56	1,29e-05

**Table 5:** Selected upregulated genes identified in periodontitis and involved in other chronic inflammatory diseases.

Ensemble ID	Gene symbol	Description	Fold change	Log_2_ fold change	*p* value	Involvement in other diseases
ENSG00000143297	FCRL5	Fc receptor-like 5	25.24	4.66	5.98e-27	Rheumatoid arthritis (RA)
ENSG00000116748	AMPD1	adenosine monophosphate deaminase 1	24.97	4.64	0.0046	Rheumatoid arthritis (RA)
ENSG00000137265	IRF4	interferon regulatory factor 4	20.10	4.33	2.31e-29	Inflammatory Bowel Disease (IBD)
ENSG00000048462	TNFRSF17	tumor necrosis factor receptor superfamily, member 17	10.43	3.38	4.80e-06	Rheumatoid arthritis (RA)
ENSG00000170866	LILRA3	leukocyte immunoglobulin-like receptor, subfamily A (without TM domain), member 3	7.45	2.90	0.008037	Rheumatoid arthritis (RA)
ENSG00000006074	CCL18	chemokine (C-C motif) ligand 18 (pulmonary and activation-regulated	6.00	2.59	1.22e-07	Rheumatoid arthritis (RA)

### The protein expression of IRF4 and CCL18 in periodontitis-affected tissue

**Figure 7:**
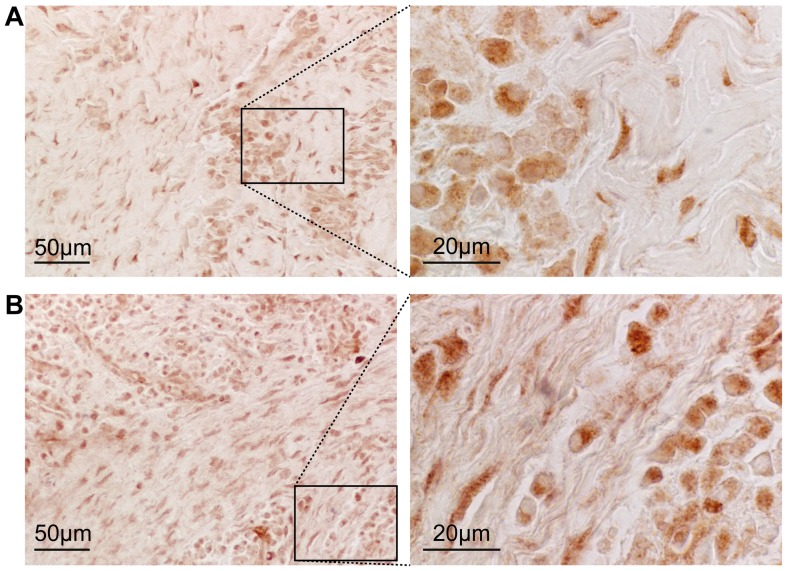
Immunohistochemical stainings of IRF4 and CCL18 in the connective tissue of periodontitis-affected gingival sections. A. Immunohistochemical staining of IRF4. B. Immunohistochemical staining of CCL18.

The expression of two of the top 50 differentially upregulated genes, IRF4 and CCL18 where further investigated at the protein level in gingival tissue samples from five additional patients with periodontitis. Immunohistochemical analysis showed that the transcription factor IRF4 and the chemokine CCL18 were expressed at the protein level in gingival tissue from patients with periodontitis (Fig. 7). IRF4 protein was expressed in cells including fibroblasts and inflammatory cells in the gingival connective tissue, as shown by morphology. For the chemokine CCL18, cellular staining of fibroblasts and inflammatory cells was observed, as well as some diffuse extracellular staining, consistent with chemokine secretion.

## Discussion

This study provides a novel quantitative comprehensive mapping of gene expression in gingival tissues from patients diagnosed with periodontitis, using RNA-Seq.

We first confirmed that the degree of inflammation was higher in periodontitis-affected gingival tissue compared to healthy tissues obtained from the same individual. Our results were based on immunohistological staining of CD3 positive cells, and further verified by RNA-Seq quantification of gene expression of the established inflammatory markers IL-1β, IL-6, IL-8, TNFα, RANTES and MCP-1. These inflammatory mediators have previously been reported to be elevated in patients with periodontitis [25], [26], [27].

Next, we performed unsupervised clustering of the gingival tissues to get an overview of the data generated from the RNA-Seq analysis. Cluster analysis revealed that the majority of periodontitis-affected clustered together and the majority of the healthy gingival tissues also clustered together, which is in line with our results regarding inflammation in the tissues. The degree of inflammation, rather than the individual, seemed to affect the clustering, indicating a common gene expression profile for periodontitis. Our results, based on the gene expression pattern of the inflammatory markers (IL-1β, IL-6, IL-8, TNFα, RANTES and MCP-1) and the immunohistochemical evaluation, confirmed that the inflammation in periodontitis involves elevated levels of locally produced cytokines in the periodontium, as has been previously demonstrated [28]. However, cluster analysis revealed that three of the patients (patient no. 6, 7 and 2) deviated from the clustering pattern. For example, the healthy gingival tissue collected from patient 6 clustered with the periodontitis-affected tissue, which could be due to moderate inflammatory infiltration (H&E score 2) observed in the healthy gingival tissue. The clustering pattern in tissue from patient 7, where the healthy and diseased gingival tissue also clustered together, could be partly explained by the patient's history of osteoarthritis, which is a disease associated with elevated levels of circulating proinflammatory cytokines IL-6 and TNFα [29]. The cluster pattern for patient 2 differed from the rest of the patient group, which could be related to this patient undergoing investigation for the inflammatory disease sarcoidosis, and in turn might affect the systemic inflammatory response. Previous studies report that oral manifestations of sarcoidosis include aggressive destruction of the periodontium with rapid periodontal bone loss [30], [31], [32]. One of these studies also emphasizes the importance of patients diagnosed with sarcoidosis to be evaluated for other systemic involvements [31]. Thus, regarding our clustering pattern, it cannot be ruled out that general health differences might have some effect on the final outcome. However, the comparison of the gene expression profiles of all individuals should minimize potential interfering signals originating from single individuals affected with other diseases.

Our RNA-Seq analysis, investigating the gene expression profile in the gingival tissues showed that the genes were differentially distributed between healthy and periodontitis-affected samples. Enrichment analysis among uniquely expressed genes in the periodontitis-affected tissues showed regulated pathways indicative of inflammation, such as cytokine signaling, chemokine signaling and the JAK-STAT signaling pathway. Several cytokines such as interleukins, which are involved in periodontits, signal through the JAK-STAT signaling pathway [33]. On the other hand, in the healthy biopsies, pathways were indicative of non-inflammatory processes that may be involved in the maintenance of the healthy gingival tissue. Future studies should also include investigation of genes within these pathways, which may contribute to understanding, prevention and treatment of periodontitis.

Differential gene expression analyses of periodontitis-affected vs. healthy gingival tissues showed the majority of differentially expressed genes to be upregulated in the periodontitis-affected tissues. Furthermore, GO enrichment analysis among these differentially expressed genes demonstrated that most of these genes were involved in immune and inflammatory processes. This is in line with the increased inflammatory response in the tissue, and also in accordance with our previous microarray studies on inflammatory-stimulated cell cultures reporting that gene expression profiles of TNFα-stimulated cells show an induction of inflammatory genes [34], [35].

Up to date, RNA-Seq studies aimed to identify new genes involved in the pathogenesis of periodontits have not been reported. One *ab initio* study by Covani et al. [36] identified genes with potential roles in periodontitis, some of which have not previously been associated with the disease. However, the protein expression of these genes in periodontitis-affected tissues has not been confirmed. In our study we aimed to identify genes involved in the pathogenesis of periodontitis. Therefore, we further searched through the differentially expressed genes, focusing on the top 50 upregulated genes. Two of these 50 upregulated genes, IRF4 and CCL18, were also detected at the protein level in periodontitis affected-tissues, supporting these genes as novel finds in the pathogenesis of periodontitis. Furthermore, these two selected genes have been reported to be involved in other chronic inflammatory diseases such as RA. The transcription factor, IRF4, has been demonstrated to be involved in T-cell-dependent chronic inflammatory diseases such as IBD [37]. Mudter et al. 2011 reported a correlation between mRNA levels of IRF4 and production of cytokines such as IL-6 and IL-17 in the inflamed colon from patients with IBD, indicating that IRF4 is involved in the regulation of chronic mucosal inflammation [37]. In addition, the gene for CCL18 was upregulated in periodontitis-affected gingival tissues. This chemokine, expressed by macrophages, monocytes and dendritic cells, has been demonstrated to be increased in synovial tissue of RA patients [38]. It has also been suggested that blockage of CCL18 expression by anti-TNF-α antibodies identifies CCL18 as an additional target for anti-TNF-α therapy in patients with RA [39], [40]. Studies are currently ongoing to investigate the expression of candidate genes novel for periodontitis in a larger cohort of patients with periodontitis and healthy controls, to be able to evaluate their impact and to further explore the possible therapeutic targeting of these genes. In addition, future studies will also be performed investigating the biological significance of the down-regulated genes in periodontitis.

In conclusion, we demonstrate for the first time, using RNA-seq, profile analysis of periodontitis revealing site-specific local variation in gene expression profiles of periodontitis-affected and healthy tissues obtained from patients diagnosed with periodontitis. Furthermore, we have identified differentially expressed novel genes in gingival tissue of periodontitis. Our findings provide a first step towards a quantitative comprehensive insight into the transcriptome of gingival tissue from patients with periodontitis, to enable identification of possible diagnostic markers of periodontitis as well as potential therapeutic targets.

## Supporting Information

Table S1 **Uniquely expressed genes within enriched pathways in periodontitis-affected and healthy gingival tissues.**
(XLSX)

Table S2 **Full list of all significantly differentially expressed genes in periodontitis-affected and healthy gingival tissues.**
(XLSX)

Table S3 **Gene Ontology enrichment analysis of differentially expressed genes.**
(XLSX)
